# The effects of continuity of care on hospital utilization in patients with knee osteoarthritis: analysis of Nationwide insurance data

**DOI:** 10.1186/s12913-018-2951-y

**Published:** 2018-03-02

**Authors:** Boyoung Jung, Kyoung Hee Cho, Dong Hyun Lee, Soyoon Kim

**Affiliations:** 1 Department of Public Health, Graduate School, Yonsei University, 50 Yonsei-ro, Seodaemun-gu, Seoul, 03722 Republic of Korea; 2 Research Department, Research Institute of Korean Medicine Policy, 91 Heojun-ro, Gangseo-gu, Seoul, 07525 Republic of Korea; 3 Asian Institute for Bioethics and Health Law (WHO Collaborating Centre for Health Law and Bioethics), Yonsei University, 50 Yonsei-ro, Seodaemun-gu, Seoul, 03722 Republic of Korea; 4 Department of International Health, Graduate school of Public Health, Yonsei University, 50 Yonsei-ro, Seodaemun-gu, Seoul, 03722 Republic of Korea

**Keywords:** Continuity of care (COC), Most frequent provider continuity (MFPC), Modified modified, Medical cost

## Abstract

**Background:**

Korea’s rapidly aging population has led to a rise in the prevalence of knee osteoarthritis (which reached upwards of 21.3% in 2017) in elderly people aged 65 years and over. Most patients with knee osteoarthritis require ongoing management in the community or through primary care. Continuity of care is a desirable attribute of primary care. However, previous studies on the association between continuity of care and health outcomes have focused on specific disease populations, particularly diabetes mellitus and hypertension. The objectives of this study were to determine whether there is an association between continuity of care for outpatients with knee osteoarthritis and health outcomes.

**Methods:**

We conducted a cohort study using claims data from 2014. The study population included 131,566 patients. We measured hospital admission and medical costs during the final 3 months and the continuity of care by Most Frequent Provider Continuity (MFPC), Modified Modified Continuity Index (MMCI), and Continuity of Care (COC) index in the 9 preceding months, using multiple logistic regression analyses to determine which index best explains continuity. We evaluated the relationship between COC and hospital admissions, using negative binomial regression analysis due to over-dispersion. Finally, multiple regressions were used to examine the relationship between the COC and medical costs.

**Results:**

We selected the COC index to determine the association between hospital admission and cost; the area under the receiver operating characteristic curve (AUC) of the COC was the largest (0.904), while those for the MFPC (0.894) and MMCI (0.893) were similar**.** The negative binomial regression analysis showed that continuity of care was significantly related to hospitalization, with the relative risk (RR) of hospital admission being low for patients with high continuity of care [RR = 27.17 for those with the reference group COC (0.76–1.00); 95% CI, 3.09–3.51]. Continuity of care was significantly related to medical costs after considering other covariates. A higher COC index was associated with a lower cost.

**Conclusions:**

Higher continuity of care for knee osteoarthritis patients might decrease hospital admission and medical costs.

**Electronic supplementary material:**

The online version of this article (10.1186/s12913-018-2951-y) contains supplementary material, which is available to authorized users.

## Background

The World Health Organization notes that rheumatic diseases are the third most important health problem in industrialized countries. Osteoarthritis is the most common, affecting 80% of the elderly population in industrialized countries [1]. Knee osteoarthritis is arguably the greatest cause of functional locomotor disability in all races and geographical areas [2, 3]. Due to population aging, arthritis affects up to 30% of those over 65 years of age [3–5] and 9.6% of men and 18% of women over 60 years of age [4]. The prevalence of painful disabling knee osteoarthritis in people over 55 years is 10%, of whom one-quarter are severely disabled [5].

Osteoarthritis of the knee is an ongoing public health problem internationally and may deteriorate with aging [6]. Many countries in Asia are rapidly aging. However, only a few population-based surveys have been conducted in Asian countries that help estimate the prevalence of osteoarthritis [6–11]. The population of Korea is rapidly aging due to the declining birthrate and increased life expectancy. The percentage of those aged 65 years or more is estimated to increase from 10.3% in 2008 to 15.6% in 2020 and 38.2% in 2050 [12]. Based on the medical statistics of medical costs in 2014, among outpatients aged 65 years or more, osteoarthritis was the fifth most common cause of hospital visits and the second most common cause of hospitalization for oriental medicine [13]. The prevalence rate in those aged 65 years or more has been continuously increasing, reaching 21.3% in 2017 [14].

Osteoarthritis is the single most common cause of disability in older adults and has a significant impact on daily life. It also tends to recur even after long-term treatment. Therefore, most patients with the condition require ongoing management in the community and through primary care [5, 15]. Continuity of care is one of the desirable attributes of primary care [16]. Previous studies have shown that fragmented visiting patterns [17–19], a shortage of primary care [19], and difficulty in accessing ambulatory care are related to preventable hospitalization [20]. In 2012, the Korean government established the chronic disease care system to effectively manage the yearly increases in chronic disease prevalence; however, this system focuses only on hypertension and diabetes [21]. Therefore, previous studies on the association between continuity of care and health outcomes have typically focused on these disease populations [22–24]. The objectives of this study were to determine whether there is an association between continuity of care for outpatients with knee osteoarthritis and two health outcomes (i.e., hospital admission and medical cost).

## Methods

### Data source

In this survey, we used data from the Korea Health Insurance Review and Evaluation Service (HIRA) 2014 National Health Insurance Card (NHIS). The NHIS includes 1.1 million patients representing the entire country (46 million patients), stratified by gender and age (5-year interval). The HIRA’s billing data are nation-based data gathered from medical institutions nationwide, equivalent to the number of claims submitted by patients. Further, data from medical assistance programs, expenditures of the government, and veteran patient claims are included in the billing data as well [25]. Data were de-identified to ensure the confidentiality of the patients. This study was approved by the HIRA Research Ethics Committee of Korea.

### Study design

We performed the analysis after modifying the cross-sectional data as a cohort. The 2014 NHIS offers cross-sectional data, which obscures the direction of the relationship between the independent and dependent variables. To overcome this limitation, we assessed the association of hospital admission in the last 3 months with continuity of care by index in the 9 preceding months. We classified patients into two groups, according to their hospital admission status and the factors influencing hospital admission. In this study, we used the conceptual model proposed by Aday [26] to analyze the factors influencing the sustainable management of joint disease. This model explains the effectiveness of prevention and treatment interventions from a clinical perspective [27], and consists of three components: structure, process, and outcome [28].

### Study population

After reviewing the most frequently seen diseases each year in traditional medicine as described previously [4], patients with the following four most frequent joint disorders were included in this study: M17 (gonarthrosis [arthrosis of the knee]), M75 (shoulder lesions), S63 (dislocation, sprain, and strain of joints and ligaments in the wrist and hand), and S93 (dislocation, sprain, and strain of joints and ligaments in the ankle and foot). Diagnoses were coded according to the 6th revision of the KCD (KCD-6), which was adapted from the International Classification of Diseases, 10th revision. Billing statements for patients with missing cost data and those with a total cost of 0 were excluded. A patient might have visited a hospital more than once during the study period (i.e., more than one claim per patient). Therefore, the number of claims in this study was higher than the number of patients. We selected patients who visited outpatient clinics with a major or secondary diagnosis code of M17 (gonarthrosis [arthrosis of knee]).

**Fig. 1:**
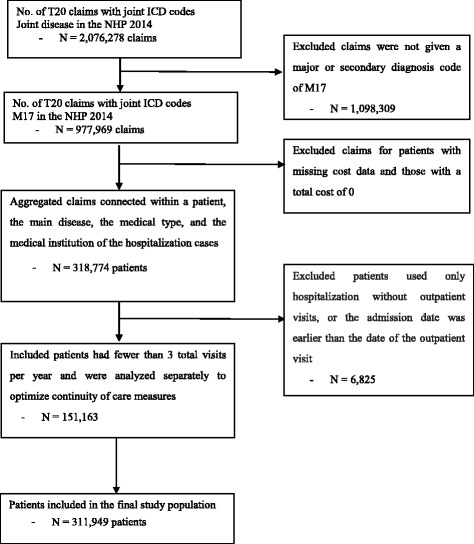
Flowchart of the selected study subjects

We excluded patients whose admission date was earlier than the date of the outpatient visit. Of the 318,774 patients with gonarthrosis, we included 311,949 patients based on their hospital admission in the last 3 months and continuity of care by index in the 9 preceding months. Finally, if the number of outpatient visits is too small, it becomes difficult to produce a meaningful level of continuity. As the number of outpatient visits increases, the degree of change in continuity level will decrease regardless of the increase in the number of outpatient medical institutions [29, 30]. For the sensitivity analysis, the subjects were retrospectively measured and analyzed, excluding patients who made fewer than 3 outpatient visits (Additional file 1). Thus, a total of 311,949 patients were ultimately included in our analysis (Fig. 1).

### Measures of study variables

#### Outcome variables

The dependent variables were hospitalization and medical expenses. Hospitalization due to knee osteoarthritis was defined by a primary or secondary diagnostic code of M17 (knee osteoarthritis) and in-hospital medical service use for more than a day. Medical expenses refer to the total self-payment expenses borne by the insured person (patient) and the amount reimbursed by the payer (Korean National Health Insurance Service) to the medical institution. The total amount of treatment items determined by the HIRA to be eligible for reimbursement is compared to that indicated in the insurance claim statement. The medical expenses of each patient refer to the total number of procedures listed in the billing record. Thereafter, the cost was converted into US dollars (US $ 1.00 = 1200 Korean won in 2016). A log conversion was performed [31] since this distribution was skewed to the left [32].

#### Measurement of continuity of care

We measured continuity of care using the Most Frequent Provider Continuity (MFPC) [33], Modified Modified Continuity Index (MMCI) [29, 34], and Continuity of Care (COC) index [35]. The MFPC, MMCI, and COC are commonly used in healthcare practice. These indexes range from 0 to 1, with higher values indicating a higher continuity of care. Each index highlights a different aspect of continuity of care. Since COC index is most commonly used among them and can reflect the number of total visits as well as the number of healthcare provider for patients, we selected COC index for analysis and classified stratified scores of COC. We divided the values into 4 groups: 0.76–1.00, 0.51–0.75, 0.26–0.50, and 0.00–0.25 using the absolute COC values. In addition, in reference to a previous study [36], three or fewer cases of outpatient use were added to one group (< 3 visits). Therefore, the final number of COC groups was 5.

$$ MFPC=\frac{\mathit{\operatorname{Max}}\left({n}_1,{n}_2\cdots, {n}_M\right)}{N} $$

$$ MMCI=\frac{1-\frac{M}{N+0.1}}{1-\frac{1}{N+0.1}} $$

$$ \mathrm{coc}=\frac{\sum_{j=1}^8{n}_j^2-N}{N\left(N-1\right)} $$

*N* = total number of visits, *n* = number of visits to provider, *js* = number of providers.

#### Other covariates

The covariates included characteristics of the primary attending hospitals, gender, age, payer type, Charlson comorbidity index (CCI) score, hospital type, region, number of owners, and the number of beds. The patient demographic data obtained from the NHI claim database included gender, age, and payer type (NHI, Medicaid, etc.). For Medicaid, an individual qualifies if the income of their household is less than $600 per month. The CCI score was measured using CCI [37], which is defined as the sum of the weights associated with each condition for which the patient can obtain claims data. The CCI scores were determined on the basis of the existence of specific ICD-10 codes in 1 year [38]. In this study, the initial CCI was defined as the CCI score of each patient. The primary attending hospital referred to the most-frequently visited medical institution for outpatient clinical practice. If the number of visits to each facility is the same, the most-recently visited medical institution was considered the primary hospital.

#### Statistical analyses

First, the demographic characteristics of the patients who were admitted to the hospital and those who were not were compared; the χ^2^ test and analysis of variance (ANOVA) were used to assess the categorical variables, and t-tests were used to assess continuous variables. Next, using a multivariable logistic regression model, we evaluated the three continuity indices (MFPC, MMCI, and COC) and hospitalization to determine which condition best explains continuity. We included the following in our model: gender, age, payer type, CCI score, hospital type, region, and ownership in 2014. Moreover, we performed both Poisson and negative binomial regression analyses (Additional file 2). When evaluating the model, the Akaike and Bayesian information criteria (AIC and BIC, respectively) are often used, where lower values indicate a better model (Additional file 3) [39]. We evaluated the relationship between COC and hospital admissions in each COC group using a negative binomial regression analysis, which was chosen due to over-dispersion. Finally, factors affecting continuity of care and the relationship between continuity of care and healthcare costs were examined through a multiple regression analysis. All statistical analyses were performed using SAS software version 9.4 (SAS, Inc., Cary, NC, USA). The results were considered statistically significant when the *p*-value was less than 0.05.

## Results

The characteristics of the study population are shown in Table 1. A total of 311,949 patients were included, of which 130,621 (41.9%) were males and 181, 328 (58.1%) were females. More than half of the participants were aged over 50 years. Of the total number of patients, 298,290 (95.6%) were registered with the NHI, and the remaining 13,413 (4.3%) were registered with Medicaid. One-third of the study population had a severe condition based on a CCI score of 3 or higher (31.7%). Most hospitals (89.1%) had fewer than 100 hospitals. In our sample, 1.8% of the patients were hospitalized and 98.2% were not hospitalized. The two groups were significantly different with respect to the individual patient characteristics.

**Table 1:** Distribution of patient characteristics by hospital admission

Unit: persons (%)								
Category		Total		No		Yes		P-value †
		N*	%	N*	%	N*	%	
Total number of patients		311,949	(100.0)	306,581	(98.2)	5368	(1.8)	
Gender	Male	130,621	(41.9)	129,002	(42.1)	1619	(30.2)	< 0.001
	Female	181,328	(58.1)	177,579	(57.9)	3749	(69.8)	
Age (yr)	≤29	69,212	(22.2)	68,928	(22.5)	284	(5.3)	< 0.001
	30–39	30,770	(9.9)	30,529	(10.0)	241	(4.5)	
	40–49	45,951	(14.7)	45,326	(14.8)	625	(11.6)	
	50–59	64,555	(20.7)	63,058	(20.6)	1497	(27.9)	
	60–69	49,299	(15.8)	47,973	(15.6)	1326	(24.7)	
	70–79	52,162	(16.7)	50,767	(16.6)	1395	(26.0)	
Payer type	NHI	298,290	(95.6)	293,263	(95.7)	5027	(93.6)	< 0.001
	Medicaid	13,413	(4.3)	13,077	(4.3)	336	(6.3)	
	Others	246	(.1)	241	(.1)	5	(.1)	
Charlson comorbidity score	0	103,063	(33.0)	102,573	(33.5)	490	(9.1)	< 0.001
	1	47,059	(15.1)	46,472	(15.2)	587	(10.9)	
	2	62,858	(20.2)	61,636	(20.1)	1222	(22.8)	
	3+	98,969	(31.7)	95,900	(31.3)	3069	(57.2)	
Frequency of hospitalization	0	306,581	(98.3)	306,581	(100.0)	0	(0.00)	< 0.001
	1	4065	(1.3)			4065	(75.7)	
	2	846	(.2)			846	(15.8)	
	3+	457	(.1)			457	(8.5)	
Outpatient visits	1	103,808	(33.3)	103,585	(33.8)	223	(4.2)	< 0.001
	2–3	79,207	(25.4)	78,701	(25.7)	506	(9.4)	
	4–7	60,431	(19.4)	59,368	(19.4)	1063	(19.8)	
	8+	68,503	(22.0)	64,927	(21.2)	3576	(66.6)	
Level of COC	0.76–1.00	78,012	(25.0)	77,519	(25.3)	493	(9.2)	< 0.001
	0.51–0.75	57,320	(18.4)	54,668	(17.8)	2652	(49.4)	
	0.26–0.50	23,534	(7.5)	21,894	(7.2)	1640	(30.6)	
	0.00–0.25	1470	(.5)	1366	(.4)	104	(1.9)	
	Low (< 3 visits)	151,613	(48.6)	151,134	(49.3)	479	(8.9)	
Hospital type	General hospital	16,730	(5.4)	15,920	(5.2)	810	(15.1)	< 0.001
	Hospital	32,457	(10.4)	30,644	(10.0)	1813	(33.8)	
	Clinic	1588	(.5)	1454	(.5)	134	(2.5)	
	LTC	175,322	(56.2)	173,278	(56.5)	2044	(38.1)	
	Oriental hospital	1725	(.6)	1663	(.5)	62	(1.2)	
	Oriental clinic	84,127	(27.0)	83,622	(27.3)	505	(9.4)	
Type of medicine	Oriental	85,852	(27.5)	85,285	(27.8)	567	(10.6)	< 0.011
	Western	225,847	(72.5)	221,050	(72.2)	4797	(89.4)	
Region	Urban	140,742	(45.1)	138,228	(45.1)	2514	(46.8)	< 0.011
	Rural	171,207	(54.9)	168,353	(54.9)	2854	(53.2)	
Ownership	Public	2282	(.7)	2216	(.7)	66	(1.2)	< 0.001
	Corporate	25,316	(8.1)	24,197	(7.9)	1119	(20.8)	
	Private	284,351	(91.2)	280,168	(91.4)	4183	(77.9)	
No. of beds	≤100	277,880	(89.1)	274,438	(89.5)	3442	(64.1)	< 0.001
	101–300	23,383	(7.5)	22,030	(7.2)	1353	(25.2)	
	301–500	4099	(1.3)	3919	(1.3)	180	(3.4)	
	501–700	2614	(.8)	2477	(.8)	137	(2.6)	
	≥701	3973	(1.3)	3717	(1.2)	256	(4.8)	

*Patient with overlapping records tallied as one patient (overlapping not allowed). †P for trend: Chi-square

*Abbreviations*: *NHI* national health insurance, *COC* continuity of care, *LTC* long-term care hospital

As shown in Table 2, individual patient and hospital characteristics had significant relations to medical costs. The table presents the frequency of hospitalizations and total medical costs, as well as the means and SDs. Further, we evaluated the association between each continuity index (MFPC, MMCI, and COC) and hospitalization to determine which index best explains continuity (Table 3). As a result, when compared to the reference group (0.76–1.00), the adjusted odds ratios of the group with a continuity level of 0.00–0.25 were 3.02 [95% CI 2.50–3.64], 2.55 [95% CI 2.10–3.10], and 10.49 [95% CI 8.18–13.46] for the MFPC, MMCI, and COC indexes, respectively. We selected the COC index to determine the association between hospital admission and cost. The area under the receiver operating characteristic curve (AUC) of the COC index was the largest (0.904), while the AUCs for the MFPC (0.894) and MMCI (0.893) were similar.

**Table 2:** Distribution of medical costs by patient characteristics

(Unit: $)										
Category		Patient	Frequency of hospitalization*				Medical costs†			
		N*	Mean	SD	Min	Max	Mean	SD	Min	Max
Gender	Male	130,621	.02	.178	0	11	127.37	538.240	0.47	139,789.78
	Female	181,328	.03	.267	0	14	202.30	638.697	1.63	28,112.14
Age (yr)	≤29	69,212	.00	.084	0	4	70.94	109.578	2.82	4624.45
	30–39	30,770	.01	.104	0	4	83.28	810.316	2.82	139,789.78
	40–49	45,951	.02	.171	0	8	110.39	248.453	1.63	10,561.95
	50–59	64,555	.03	.263	0	11	171.71	450.686	0.47	24,586.82
	60–69	49,299	.04	.286	0	10	265.98	775.475	2.19	28,112.14
	70–79	52,162	.04	.349	0	14	317.80	907.321	2.19	24,694.04
Payer type	NHI	298,290	.02	.222	0	10	165.96	591.144	0.47	139,789.78
	Medicaid	13,413	.05	.424	0	14	279.46	760.654	2.19	24,586.82
	Others	246	.03	.237	0	2	273.59	539.213	5.68	5011.12
Charlson comorbidity score	0	103,063	.01	.085	0	4	72.69	117.522	2.82	6607.16
	1	47,059	.02	.156	0	8	112.84	683.632	1.63	139,789.78
	2	62,858	.03	.218	0	11	158.13	356.459	0.47	12,885.72
	3+	98,969	.05	.350	0	14	308.96	886.541	2.19	28,112.14
Outpatient visits	1	103,808	.00	.099	0	9	31.54	115.540	0.47	15,756.91
	2–3	79,207	.01	.148	0	14	68.70	186.207	4.38	13,888.52
	4–7	60,431	.02	.236	0	11	152.88	444.964	3.92	28,112.14
	8+	68,503	.07	.396	0	14	516.26	1114.627	13.75	139,789.78
Level of COC	0.76–1.00	78,012	.01	.106	0	10	170.09	312.047	3.92	24,586.82
	0.51–0.75	57,320	.07	.384	0	14	387.94	1040.586	17.70	139,789.78
	0.26–0.50	1470	.13	.615	0	11	202.20	627.165	32.03	9973.78
	0.00–0.25	23,534	.10	.454	0	9	489.31	1157.925	17.89	19,840.50
	Low (< 3 visits)	151,613	.00	.115	0	14	39.58	134.471	0.47	15,756.91
Hospital type	General Hospital	16,730	.07	.372	0	14	281.58	1082.456	2.19	28,112.14
	Hospital	32,457	.07	.351	0	10	228.84	776.606	2.19	16,298.44
	Clinic	1588	.35	1.421	0	14	333.70	1282.782	2.19	16,734.24
	LTC	175,322	.02	.180	0	9	163.87	584.362	0.47	139,789.78
	Oriental Hospital	1725	.07	.453	0	8	173.74	395.500	5.95	7899.90
	Oriental Clinic	84,127	.01	.121	0	8	138.15	342.284	5.95	14,081.61
Region	Urban	140,742	.03	.239	0	14	173.88	660.958	0.47	139,789.78
	Rural	171,207	.02	.230	0	11	168.49	544.449	1.63	28,112.14
Ownership	Public	2282	.04	.296	0	6	253.48	1145.142	3.30	14,281.70
	Corporate	25,316	.07	.441	0	14	253.91	930.462	2.19	28,112.14
	Private	284,351	.02	.205	0	10	162.87	553.520	0.47	139,789.78
No. of beds	≤100	277,880	.02	.189	0	14	159.10	532.764	0.47	139,789.78
	101–300	23,383	.09	.460	0	11	246.28	894.096	2.19	28,112.14
	301–500	4099	.07	.426	0	9	226.00	822.218	2.19	19,486.28
	501–700	2614	.09	.547	0	14	348.73	1320.391	4.40	24,694.04
	≥701	3973	.09	.381	0	5	380.78	1318.467	5.01	19,840.50

*Calculated total number of hospital admissions divided into total of person–years

† The sum of self-payment costs paid by the beneficiary (patient) and benefits reimbursed by the insurer (Korean National Health Insurance Service) to the medical care institution. The total amount of treatment items determined to be eligible for reimbursement by the HIRA is compared to the amount indicated in the submitted insurance claim statement. Costs are in Korean Won (1200 KRW = 1 US dollar)

*Abbreviations*: *SD* standard deviation; *HIRA* Health Insurance Review Assessment Service

**Table 3:** Odds ratios (ORs) and areas under the curve (AUCs) for hospitalization by continuity index

Index	No		Yes		Total	*P*-value†	Unadjusted OR				Adjusted OR^a^				AUC^b^
	N*	%	N*	%											
Total	306,581	98.3	5368	1.7	311,949		OR	95% CI		*P*-value	OR	95% CI		*P*-value	
Most Frequent Provider Continuity (MFPC)															
0.76–1.00	106,237	98.3	1892	1.7	108,129	< 0.001	1.00				1.00				0.894
0.51–0.75	31,733	95.2	1600	4.8	33,333		5.62	5.08	6.21	< 0.0001	0.97	0.81	1.17	0.781	
0.26–0.50	17,135	92.7	1345	7.3	18,480		15.91	14.35	17.63	< 0.0001	2.38	1.98	2.85	< 0.0001	
0.00–0.25	342	86.8	52	13.2	394		24.77	22.29	27.52	< 0.0001	3.02	2.50	3.64	< 0.0001	
< 3 visits	151,134	99.7	479	0.3	151,613		47.97	35.36	65.10	< 0.0001	5.51	3.81	7.98	< 0.0001	
Modified Modified Continuity Index (MMCI)															
0.76–1.00	120,159	97.5	3144	2.5	123,303	< 0.001	1.00				1.00				0.893
0.51–0.75	21,436	94.9	1157	5.1	22,593		8.26	7.50	9.09	< 0.0001	0.58	0.48	0.71	< 0.0001	
0.26–0.50	10,656	96.8	349	3.2	11,005		17.03	15.30	18.96	< 0.0001	1.33	1.09	1.64	0.006	
0.00–0.25	3196	93.0	239	7.0	3435		10.33	8.99	11.88	< 0.0001	3.02	2.50	3.64	< 0.0001	
< 3 visits	151,134	99.7	479	0.3	151,613		23.59	20.12	27.66	< 0.0001	5.28	4.21	6.61	< 0.0001	
Continuity of Care (COC)															
0.76–1.00	77,519	99.4	493	0.6	78,012	< 0.001	1.00				1.00				0.904
0.51–0.75	54,668	95.4	2652	4.6	57,320		2.01	1.77	2.28	< 0.0001	0.46	0.38	0.57	< 0.0001	
0.26–0.50	21,894	93.0	1640	7.0	23,534		15.31	13.88	16.88	< 0.0001	2.55	2.10	3.10	< 0.0001	
0.00–0.25	1366	92.9	104	7.1	1470		24.02	19.30	29.89	< 0.0001	10.49	8.18	13.46	< 0.0001	
< 3 visits	151,134	99.7	479	0.3	151,613		23.63	21.33	26.19	< 0.0001	3.70	3.02	4.54	< 0.0001	

*Patient with overlapping records tallied as one patient (overlapping not allowed)

†P for trend: Chi-square. ^a^Odds ratio and 95% confidence intervals (CIs) were calculated from multiple logistic regression models

It was adjusted by each continuity index (MFPC, MMCI, and COC) separately and all other independent variables due to multicollinearity between index

^b^AUC (area under the receiver operating characteristic curve) means discrimination ability of the prediction model. It ranges from 0.5 to 1, with 1 indicating perfect discrimination

Table 4 shows the adjusted relative risk (RR) at admission. After controlling for all covariates, we calculated the adjusted RR of the COC index group (based on the 0.00–0.25 group). The results showed that the COC index group of 0.76–1.00 COC index group had RR of 27.17 (95% CI 3.09 to 3.51), whereas the RRs was 0.26–0.50 and 0.51–0.75 index groups had RRs of 8.56 (95% CI 2.05 to 2.24) and 6.03 (95% CI 1.71 to 1.89), respectively. However, in the low group (< 3 visits), the adjusted RR for inpatient hospitalization was less than 1.00 (RR = 0.98, 95% CI 0.80–1.15). Therefore, negative binomial regression analysis showed that the continuity of care was significantly associated with hospitalization, and the hospitalization rate of patients with high hospital stay was low.

**Table 4:** Relative risk for hospital admission calculated using a negative binomial regression model

Category	Unadjusted				Adjusted^a^			
	RR	95% CI		*P*-value	RR	95% CI		*P*-value
Gender								
Male	1.00				1.00			
Female	1.88	0.58	0.68	< 0.0001	1.24	0.16	0.27	< 0.0001
Age (yr)								
≤29	1.00				1.00			
30–39	1.77	0.41	0.73	< 0.0001	1.51	0.24	0.58	< 0.0001
40–49	3.51	1.13	1.38	< 0.0001	1.94	0.96	0.36	< 0.0001
50–59	6.52	1.76	1.99	< 0.0001	4.56	1.85	1.18	< 0.0001
60–69	7.77	1.94	2.17	< 0.0001	14.04	2.99	2.29	< 0.0001
≥70	8.89	2.07	2.30	< 0.0001	13.72	2.97	2.27	< 0.0001
Payer type								
National health insurance	1.00				1.00			
Medicaid	2.00	0.61	0.78	< 0.0001	1.13	0.03	0.21	0.013
Others	1.38	0.39	1.02	0.375	1.98	0.07	1.44	0.075
Hospital type								
General hospital	8.41	2.03	2.23	< 0.0001	12.50	2.40	2.65	< 0.0001
Hospital	9.04	2.12	2.29	< 0.0001	11.50	2.35	2.53	< 0.0001
Clinic	43.32	3.65	3.89	< 0.0001	57.54	3.91	4.20	< 0.0001
Long-term care hospital	2.04	0.63	0.80	< 0.0001	2.00	0.61	0.78	< 0.0001
Oriental hospital	8.67	1.96	2.36	< 0.0001	9.56	2.04	2.48	< 0.0001
Oriental clinic	1.00				1.00			
Region								
Urban	1.00				1.00			
Rural	0.94	0.10	0.01	0.010	0.99	0.06	0.04	0.643
Ownership								
Public	1.00				1.00			
Corporate	1.60	0.26	0.68	< 0.0001	1.69	0.30	0.75	< 0.0001
Private	0.47	0.96	0.56	< 0.0001	1.72	0.31	0.77	< 0.0001
COC								
0.76–1.00	1.00				1.00			
0.51–0.75	8.88	2.10	2.27	< 0.0001	6.03	1.71	1.89	< 0.0001
0.26–0.50	13.50	2.51	2.69	< 0.0001	8.56	2.05	2.24	< 0.0001
0.00–0.25	17.36	2.68	3.03	< 0.0001	27.17	3.09	3.51	< 0.0001
Low (< 3 visits)	0.64	0.55	0.33	< 0.0001	2.66	0.80	1.15	< 0.0001
Deviance/df					1.089			
LL					− 5667.230			
LL χ^2^					799.779			
AIC					11,378.461			
BIC					11,523.402			

^a^Fitness information of the adjusted model

*Abbreviations*: *LL* likelihood ratio, *AIC* akaike information criterion, *BIC* bayesian information criterion

The multiple regression analysis revealed a relationship between various patient characteristics and medical costs. In terms of gender, the cost of medicine was higher for women than for men (β = 0.044). Furthermore, there was an increase in medical expenses as age increased. Continuity of care was shown to have a significant effect on medical costs after considering other covariates. For the 0.76–1.00 COC index group, the cost was significantly lower than was that for the 0.00–0.25 COC index group. Therefore, higher COC indices were associated with lower costs (Table 5).

**Table 5:** Coefficients and standard errors calculated by multiple linear regression analysis

Category	Unadjusted			Adjusted^a^		
	Coefficient	SE	*P*-value	Coefficient	SE	*P*-value
Gender						
Male						
Female	0.283	0.004	< 0.0001	0.044	0.003	< 0.0001
Age (yr)						
≤ 29						
30–39	−0.010	0.008	0.185	−0.073	0.005	< 0.0001
40–49	0.189	0.007	< 0.0001	−0.060	0.005	< 0.0001
50–59	0.469	0.006	< 0.0001	0.011	0.004	0.008
60–69	0.751	0.006	< 0.0001	0.096	0.005	< 0.0001
≥ 70	0.877	0.006	< 0.0001	0.129	0.005	< 0.0001
Payer type						
National health insurance						
Medicaid	0.414	0.010	< 0.0001	0.174	0.007	< 0.0001
Others	0.599	0.073	< 0.0001	0.220	0.048	< 0.0001
Hospital type						
General hospital	0.088	0.011	< 0.0001	0.072	0.010	< 0.0001
Hospital	0.028	0.030	0.349	0.204	0.020	< 0.0001
Clinic	0.151	0.009	< 0.0001	0.262	0.012	< 0.0001
LTC	0.062	0.029	0.033	0.180	0.021	< 0.0001
Oriental hospital	0.158	0.010	< 0.0001	0.361	0.012	< 0.0001
Oriental clinic						
Region						
Urban	0.038	0.004	< 0.0001	0.031	0.003	< 0.0001
Rural						
Ownership						
Public						
Corporate	0.204	0.025	< 0.0001	0.219	0.016	< 0.0001
Private	0.091	0.024	< 0.0001	0.271	0.016	< 0.0001
COC						
0.76–1.00						
0.51–0.75	−1.348	0.003	< 0.0001	−0.136	0.020	< 0.0001
0.26–0.50	−0.134	0.020	< 0.0001	−1.333	0.003	< 0.0001
0.00–0.25	0.692	0.006	< 0.0001	0.677	0.006	< 0.0001
Low (< 3 visits)	0.618	0.004	< 0.0001	0.608	0.004	< 0.0001

^a^The Adj R-Sq of model was 0.583

*Abbreviations*: *SE* standard error

## Discussion

We are using a nationwide group-based approach to explore the relationship between continuity of care and health outcomes (hospitalization and medical expenses) of patients with knee osteoarthritis in Korea. Our findings show that higher rates of continuous care reduced hospitalization and medical expenses. These results agree with those of previous studies, showing the association between patient continuity and outcomes [29, 40, 41], which suggest that improved continuity of care may save costs [42, 43].

Previous studies have identified possible mechanisms for these findings. A comprehensive review of care continuity suggested that three types of continuity—information, management, and relational—exist in all settings [44]. In another study, it was found that both the concept and measurement of continuity of primary care and the relationship between patients and physicians are important [45], and that these affect health outcomes [46]. Continuity of care can only be achieved by bridging the individual elements of the care pathway until the underlying mechanism by which the care is provided is understood. In previous studies, Sirski and Dowden [47, 48] found that those suffering chronic disease are more likely to use outpatient services than healthy individuals and tend to establish relationships with physicians more rapidly. We discovered that the benefits of this technology may be expanded among chronic disease patients.

The NHI in Korea, based on the principle of universal coverage, has improved people’s access to medical care. Furthermore, South Korea’s geography is favorable because its land area is relatively small and it has reliable travel between regions [49]. -As access to medical care improves, the pattern of medical use is an attempt to reduce preventable hospitalizations. In this way, the managed care delivery system in Korea is different from the United States, where the selection of patients’ healthcare providers is restricted and regulated [50]. Specifically, primary care physicians in Korea work primarily with independent private practices and are reimbursed for each service. This system allows patients to select and continue to see a specific doctor, regardless of changes in their employment status. Korea’s society is aging faster than any other country. As the number of elderly people increases, so do the medical expenses for chronic degenerative diseases, and the social burden increases. If elderly people with knee joint issues, for example, maintain continuity of care, they can avoid unnecessary hospitalizations and the accompanying medical expenses. In order to analyze the extent to which continuous management reduces the burden of admission and medical expenses in Korea, the results of estimating the difference in medical burden between the highest and lowest sustainability indicators are shown in Additional file 4.

This study has some limitations. We could not take all the factors into consideration affecting continuity of care and health outcomes because not all factors were included in the billing data. According to previous studies, continuity of care is based on the characteristics of the healthcare provider (e.g., the age and sex of the doctor, whether they are general or specialist, medical care period) or the patient (e.g. income level, educational level, residence, health care satisfaction) [51]. When analyzing the relationship between nursing care continuity and health outcomes, the residential area was considered to be a confusing variable; therefore, the hospital’s area was set as the residential area [52]. In addition, the income variable of our study used the payer type (NHI/Medicaid) as an agent. In Korea, Medicaid is a social security system that provides basic medical services to people with incomes falling below a certain threshold [49]. Medicaid recipients thus belong to a socioeconomically lower class with corresponding higher probabilities of comorbidity. The lower continuity of the Medicaid patients may have been the result of these characteristics [51]. Certainly, this is in line with previous studies, which have shown that Medicaid recipients have a lower level of continuity than NHI beneficiaries [53]. Our results show that Medicaid patients tend to be hospitalized more than health insurance members [54, 55]. To establish other possible factors, it is necessary to conduct a qualitative survey using questionnaires and interviews with patients and healthcare providers.

Based on the information in the billing database, we calculated only direct medical expenses. Generally, it takes time to calculate non-medical expenses such as transportation costs and productivity decreases due to musculoskeletal disease morbidity [56]. With musculoskeletal conditions, the indirect costs (productivity and loss of wages) tend to be much greater than direct expenses [57]. In the United States and Canada, for example, this corresponds to 2.4% and 1.3% of the gross national product, respectively [58, 59]. Since the billing data contained only information on the medical service provided, the medical expenses for this survey did not include uncovered areas, such as treatment to alleviate physical distress. However, despite the lack of data, it was still possible to investigate whether healthcare costs decreased as the continuity of care increased in this study. This is similar to Raddish et al. [42].

Finally, there was a problem with accurate diagnoses, due to the nature of the billing data. In other words, these data are collected not for clinical purposes [25] but for refunding medical services. Fleming et al. [60] and Godkin and Rice [61] reported that there was a difference in continuity according to the condition, and continuity was clearly higher for those with chronic diseases. The accuracy of diagnosis in NHI’s assertion data is estimated to be about 70% [62]. Therefore, the effectiveness of diagnosis may an issue for our research. We examined not only the major diagnostic codes but also secondary codes to improve diagnostic accuracy.

Despite the limitations noted, our research has several advantages. First, national claims data was used to analyze representative samples of patients with knee joint disease. Subjects were all Korean patients who had been diagnosed with knee joint disease in 2014. Secondly, by using cross-sectional data that was representative of the whole country, the model made it possible to identify causal relations in the study subjects. Since many previous studies have been cross-sectional, using claims data from 1 or 2 years, they have had limitations in their ability to prove causality [29]. Given this validity problem, we recommend that research using a management database such as the Korea Health Insurance Application Database should focus specifically on patients who had at least three outpatient clinic visits during the study period (Additional file 1). Third, unlike previous studies of diabetes and hypertension, knee joint disease was analyzed. This study is rare in that it confirms the relevance between care continuity and health outcomes in individuals with knee joint disease. More effort is needed to quantify the quality of management so as to provide supportive evidence to expand the coverage of the Chronic Disease Care System. Although continuity of care is a desirable attribute for those with chronic illnesses, little research on health services has considered this relationship. Future examinations could extend the information now available for knee joint diseases to other chronic diseases.

## Conclusions

In conclusion, this study suggests that the risk of hospitalization and health care costs decreases when patients focus on using the same healthcare provider. Furthermore, in the aging society of Korea, it has been shown that improving the continuity of care is a good strategy to improve the quality of health care for the elderly and promote efficient expenditure of medical budgets. Therefore, policymakers need to improve the continuity of care for elderly patients with knee joint disease.

## Additional files

Additional file 1 Relative risk for hospital admission, calculated using a negative binomial regression model according to inclusion of individuals with < 3 visits (DOCX 29 kb)

Additional file 2 Relative risk for hospital admission, calculated using a Poisson regression model (DOCX 28 kb)

Additional file 3 Relative risk for hospital admission, calculated using a Poisson regression model and negative binomial regression model (DOCX 32 kb)

Additional file 4 Reduction in hospitalization and medical costs, according to the level of continuity of care (DOCX 19 kb)

## Abbreviations

AUROC: Area under the receiver operating curve
COC: Continuity of care
HIRA: Health Insurance Review & Assessment Services
MFPC: Most frequent provider continuity
MMCI: Modified modified continuity index
NHIS: National Health Insurance Sample
