# Supplementary material for: The effects of continuity of care on hospital utilization in patients with knee osteoarthritis: analysis of Nationwide insurance data
Source: BMC Health Serv Res. 2018 Mar 2;18:152. doi: 10.1186/s12913-018-2951-y (PMC5833114; doi:10.1186/s12913-018-2951-y)
Supplement: Supplementary file 1 — Relative risk for hospital admission, calculated using a negative binomial regression model according to inclusion of individuals with < 3 visits (DOCX 29 kb) [file 12913_2018_2951_MOESM1_ESM.docx]

**- File name: Additional file 1**

**- Title of data:** Relative risk for hospital admission, calculated using a negative binomial regression model according to inclusion of individuals with < 3 visits

| Category | Excluding those with <3 visits  (Negative binominal regression model) | | | | | |  | Including those with <3 visits  (Negative binominal regression model) | | | | |
| --- | --- | --- | --- | --- | --- | --- | --- | --- | --- | --- | --- | --- |
|  | | RR | 95% CI | | P-value |  | | | RR | 95% CI | | P-value |
| Gender | |  |  |  |  |  | | |  |  |  |  |
| Male | | 1.00 |  |  |  |  | | | 1.00 |  |  |  |
| Female | | 1.29 | 0.19 | 0.31 | <0.0001 |  | | | 1.24 | 0.16 | 0.27 | <0.0001 |
| Age (yr) | |  |  |  |  |  | | |  |  |  |  |
| ≤29 | | 1.00 |  |  |  |  | | | 1.00 |  |  |  |
| 30–39 | | 1.46 | 0.19 | 0.56 | <0.0001 |  | | | 1.51 | 0.24 | 0.58 | <0.0001 |
| 40–49 | | 1.76 | 0.88 | 0.25 | <0.0001 |  | | | 1.94 | 0.96 | 0.36 | <0.0001 |
| 50–59 | | 4.20 | 1.79 | 1.08 | <0.0001 |  | | | 4.56 | 1.85 | 1.18 | <0.0001 |
| 60–69 | | 12.84 | 2.92 | 2.19 | <0.0001 |  | | | 14.04 | 2.99 | 2.29 | <0.0001 |
| ≥70 | | 13.09 | 2.94 | 2.20 | <0.0001 |  | | | 13.72 | 2.97 | 2.27 | <0.0001 |
| Payer type | |  |  |  |  |  | | |  |  |  |  |
| NIC | | 1.00 |  |  |  |  | | | 1.00 |  |  |  |
| Medicaid | | 1.16 | 0.05 | 0.25 | 0.003 |  | | | 1.13 | 0.03 | 0.21 | 0.013 |
| Others | | 0.51 | 1.50 | 0.16 | 0.112 |  | | | 1.98 | 0.07 | 1.44 | 0.075 |
| Hospital type | |  |  |  |  |  | | |  |  |  |  |
| General hospital | | 10.73 | 2.25 | 2.49 | 1508.520 |  | | | 12.50 | 2.40 | 2.65 | <0.0001 |
| Hospital | | 10.58 | 2.27 | 2.45 | 2564.603 |  | | | 11.50 | 2.35 | 2.53 | <0.0001 |
| Clinic | | 45.57 | 3.72 | 4.01 | 2728.683 |  | | | 57.54 | 3.91 | 4.20 | <0.0001 |
| LTC | | 2.03 | 0.62 | 0.79 | 262.261 |  | | | 2.00 | 0.61 | 0.78 | <0.0001 |
| Oriental hospital | | 8.93 | 1.97 | 2.41 | 385.102 |  | | | 9.56 | 2.04 | 2.48 | <0.0001 |
| Oriental clinic | | 1.00 |  |  |  |  | | | 2.72 |  |  |  |
| Region | |  |  |  |  |  | | |  |  |  |  |
| Urban | | 1.00 |  |  |  |  | | | 1.00 |  |  |  |
| Rural | | 1.00 | 0.05 | 0.05 | 0.995 |  | | | 0.99 | 0.06 | 0.04 | 0.643 |
| Ownership | |  |  |  |  |  | | |  |  |  |  |
| Public | | 1.00 |  |  |  |  | | | 1.00 |  |  |  |
| Corporation | | 1.61 | 0.24 | 0.71 | <0.0001 |  | | | 1.69 | 0.30 | 0.75 | <0.0001 |
| Private | | 1.61 | 0.24 | 0.71 | <0.0001 |  | | | 1.72 | 0.31 | 0.77 | <0.0001 |
| COC | |  |  |  |  |  | | |  |  |  |  |
| 0.76–1.00 | | 1.00 |  |  |  |  | | | 1.00 |  |  |  |
| 0.51–0.75 | | 8.05 | 2.00 | 2.18 | <0.0001 |  | | | 6.03 | 1.71 | 1.89 | <0.0001 |
| 0.26–0.50 | | 11.92 | 2.38 | 2.57 | <0.0001 |  | | | 8.56 | 2.05 | 2.24 | <0.0001 |
| 0.00–0.25 | | 13.46 | 2.42 | 2.78 | <0.0001 |  | | | 27.17 | 3.09 | 3.51 | <0.0001 |
| Low (<3 visits) | |  |  |  |  |  | | | 2.66 | 0.80 | 1.15 | <0.0001 |
| Deviance/df | | 1.108 | | | |  | | | 1.089 | | | |

Abbreviations: LTC, long-term care hospital; COC, continuity of care; RR, relative risk; CI, confidence interval

**- Description of data:**

Given the validity problem mentioned earlier, we recommend that studies using administrative databases, such as the Korea National Health Insurance Claims Database, should focus specifically on patients who have three or more outpatient care visits during the study period. For sensitivity analysis, the participants were measured and analyzed retrospectively, except for patients who had fewer than three outpatient visits. Better figures for deviance/df were seen for patients who had fewer than three visits.
