# Supplementary material for: The effects of continuity of care on hospital utilization in patients with knee osteoarthritis: analysis of Nationwide insurance data
Source: BMC Health Serv Res. 2018 Mar 2;18:152. doi: 10.1186/s12913-018-2951-y (PMC5833114; doi:10.1186/s12913-018-2951-y)
Supplement: Supplementary file 2 — Relative risk for hospital admission, calculated using a Poisson regression model (DOCX 28 kb) [file 12913_2018_2951_MOESM2_ESM.docx]

**- File name: Additional file 2**

**- Title of data:** Relative risk for hospital admission, calculated using a Poisson regression model

| Category | Unadjusted RR (Poisson regression) | | | |  | Adjusted RR (Poisson regression) | | | |
| --- | --- | --- | --- | --- | --- | --- | --- | --- | --- |
|  | RR | 95% CI | | P-value |  | RR | 95% CI | | P-value |
| Gender |  |  |  |  |  |  |  |  |  |
| Male | 1.00 |  |  |  |  | 1.00 |  |  |  |
| Female | 1.88 | 0.58 | 0.68 | <0.0001 |  | 1.29 | 0.20 | 0.30 | <0.0001 |
| Age (yr) |  |  |  |  |  |  |  |  |  |
| ≤29 | 1.00 |  |  |  |  | 1.00 |  |  |  |
| 30–39 | 1.77 | 0.41 | 0.73 | <0.0001 |  | 1.04 | 0.10 | 0.02 | 0.198 |
| 40–49 | 3.51 | 1.13 | 1.38 | <0.0001 |  | 2.79 | 0.94 | 1.11 | <0.0001 |
| 50–59 | 6.52 | 1.76 | 1.99 | <0.0001 |  | 6.35 | 1.68 | 2.01 | <0.0001 |
| 60–69 | 7.77 | 1.94 | 2.17 | <0.0001 |  | 19.26 | 2.66 | 3.25 | <0.0001 |
| ≥70 | 8.89 | 2.07 | 2.30 | <0.0001 |  | 12.74 | 2.25 | 2.84 | <0.0001 |
| Payer type |  |  |  |  |  |  |  |  |  |
| NHI | 1.00 |  |  |  |  | 1.00 |  |  |  |
| Medicaid | 2.00 | 0.61 | 0.78 | <0.0001 |  | 1.20 | 0.10 | 0.26 | <0.0001 |
| Others | 1.38 | 0.37 | 1.01 | 0.367 |  | 2.04 | 1.41 | 0.01 | 0.045 |
| Hospital type |  |  |  |  |  |  |  |  |  |
| General hospital | 8.41 | 2.03 | 2.22 | <0.0001 |  | 11.61 | 2.34 | 2.56 | <0.0001 |
| Hospital | 9.04 | 2.12 | 2.29 | <0.0001 |  | 10.90 | 2.30 | 2.48 | <0.0001 |
| Clinic | 43.32 | 3.66 | 3.88 | <0.0001 |  | 38.89 | 3.54 | 3.78 | <0.0001 |
| Long-term care hospital | 2.04 | 0.63 | 0.79 | <0.0001 |  | 2.00 | 0.61 | 0.78 | <0.0001 |
| Oriental hospital | 8.67 | 1.97 | 2.35 | <0.0001 |  | 8.98 | 2.00 | 2.39 | <0.0001 |
| Oriental clinic | 1.00 |  |  |  |  | 1.00 |  |  |  |
| Region |  |  |  |  |  |  |  |  |  |
| Urban | 1.00 |  |  |  |  | 1.00 |  |  |  |
| Rural | 0.94 | 0.10 | 0.01 | 0.009 |  | 1.03 | 0.02 | 0.07 | 0.285 |
| Ownership |  |  |  |  |  |  |  |  |  |
| Public | 1.00 |  |  |  |  | 1.00 |  |  |  |
| Corporation | 1.60 | 0.27 | 0.67 | <0.0001 |  | 1.60 | 0.27 | 0.67 | <0.0001 |
| Private | 0.47 | 0.96 | 0.56 | <0.0001 |  | 1.60 | 0.27 | 0.68 | <0.0001 |
| COC |  |  |  |  |  |  |  |  |  |
| 0.76–1.00 | 1.00 |  |  |  |  | 1.00 |  |  |  |
| 0.51–0.75 | 8.88 | 2.10 | 2.27 | <0.0001 |  | 5.94 | 1.69 | 1.87 | <0.0001 |
| 0.26–0.50 | 13.50 | 2.51 | 2.69 | <0.0001 |  | 8.06 | 1.99 | 2.18 | <0.0001 |
| 0.00–0.25 | 17.36 | 2.69 | 3.02 | <0.0001 |  | 27.65 | 3.14 | 3.50 | <0.0001 |
| Low (<3 visits) | 0.64 | 0.55 | 0.34 | <0.0001 |  | 2.66 | 0.81 | 1.15 | <0.0001 |

**- Description of data:**

Because of the dependent variable being a countable variable, we performed both Poisson and Negative binomial regression analyses. Therefore, we have included a negative binomial regression model in Table 4 and suggest a Poisson model in Additional File 2.
