# Supplementary material for: The effects of continuity of care on hospital utilization in patients with knee osteoarthritis: analysis of Nationwide insurance data
Source: BMC Health Serv Res. 2018 Mar 2;18:152. doi: 10.1186/s12913-018-2951-y (PMC5833114; doi:10.1186/s12913-018-2951-y)
Supplement: Supplementary file 3 — Relative risk for hospital admission, calculated using a Poisson regression model and negative binomial regression model (DOCX 32 kb) [file 12913_2018_2951_MOESM3_ESM.docx]

| **- File name: Additional file 3**  **- Title of data:** Relative risk for hospital admission, calculated using a Poisson regression model and negative binomial regression model | | | | | | | | | | | |  |
| --- | --- | --- | --- | --- | --- | --- | --- | --- | --- | --- | --- | --- |
| Category | Poisson regression model | | | |  | | Negative binominal regression model | | | | |  |
|  | Relative risk (95% CI) | | | |  | | Relative risk (95% CI) | | | | |  |
|  | RR | 95% CI | | P-value | |  | | RR | 95% CI | | P-value | |
| Gender |  |  |  |  | |  | |  |  |  |  | |
| Male | 1.00 |  |  |  | |  | | 1.00 |  |  |  | |
| Female | 1.29 | 0.20 | 0.30 | <0.0001 | |  | | 1.24 | 0.16 | 0.27 | <0.0001 | |
| Age (yr) |  |  |  |  | |  | |  |  |  |  | |
| ≤29 | 1.00 |  |  |  | |  | | 1.00 |  |  |  | |
| 30–39 | 1.04 | 0.10 | 0.02 | 0.198 | |  | | 1.51 | 0.24 | 0.58 | <0.0001 | |
| 40–49 | 2.79 | 0.94 | 1.11 | <0.0001 | |  | | 1.94 | 0.96 | 0.36 | <0.0001 | |
| 50–59 | 6.35 | 1.68 | 2.01 | <0.0001 | |  | | 4.56 | 1.85 | 1.18 | <0.0001 | |
| 60–69 | 19.26 | 2.66 | 3.25 | <0.0001 | |  | | 14.04 | 2.99 | 2.29 | <0.0001 | |
| ≥70 | 12.74 | 2.25 | 2.84 | <0.0001 | |  | | 13.72 | 2.97 | 2.27 | <0.0001 | |
| Payer type |  |  |  |  | |  | |  |  |  |  | |
| NHI | 1.00 |  |  |  | |  | | 1.00 |  |  |  | |
| Medicaid | 1.20 | 0.10 | 0.26 | <0.0001 | |  | | 1.13 | 0.03 | 0.21 | 0.013 | |
| Others | 2.04 | 0.01 | 1.41 | 0.045 | |  | | 1.98 | 0.07 | 1.44 | 0.075 | |
| Hospital type |  |  |  |  | |  | |  |  |  |  | |
| General hospital | 11.61 | 2.34 | 2.56 | <0.0001 | |  | | 12.50 | 2.40 | 2.65 | <0.0001 | |
| Hospital | 10.90 | 2.30 | 2.48 | <0.0001 | |  | | 11.50 | 2.35 | 2.53 | <0.0001 | |
| Clinic | 38.89 | 3.54 | 3.78 | <0.0001 | |  | | 57.54 | 3.91 | 4.20 | <0.0001 | |
| LTC | 2.00 | 0.61 | 0.78 | <0.0001 | |  | | 2.00 | 0.61 | 0.78 | <0.0001 | |
| Oriental hospital | 8.98 | 2.00 | 2.39 | <0.0001 | |  | | 9.56 | 2.04 | 2.48 | <0.0001 | |
| Oriental clinic | 1.00 |  |  |  | |  | | 1.00 |  |  |  | |
| Region |  |  |  |  | |  | |  |  |  |  | |
| Urban | 1.00 |  |  |  | |  | | 1.00 |  |  |  | |
| Rural | 1.03 | 0.02 | 0.07 | 0.285 | |  | | 0.09 | 0.06 | 0.04 | 0.643 | |
| Ownership |  |  |  |  | |  | |  |  |  |  | |
| Public | 1.00 |  |  |  | |  | | 1.00 |  |  |  | |
| Corporation | 1.60 | 0.27 | 0.67 | <0.0001 | |  | | 1.69 | 0.30 | 0.75 | <0.0001 | |
| Private | 1.60 | 0.27 | 0.68 | <0.0001 | |  | | 1.72 | 0.31 | 0.77 | <0.0001 | |
| COC |  |  |  |  | |  | |  |  |  |  | |
| 0.76–1.00 | 1.00 |  |  |  | |  | | 1.00 |  |  |  | |
| 0.51–0.75 | 5.94 | 1.69 | 1.87 | <0.0001 | |  | | 6.03 | 1.71 | 1.89 | <0.0001 | |
| 0.26–0.50 | 8.06 | 1.99 | 2.18 | <0.0001 | |  | | 8.56 | 2.05 | 2.24 | <0.0001 | |
| 0.00–0.25 | 27.65 | 3.14 | 3.50 | <0.0001 | |  | | 27.17 | 3.09 | 3.51 | <0.0001 | |
| Low (<3 visits) | 2.66 | 0.81 | 1.15 | <0.0001 | |  | | 2.66 | 0.80 | 1.15 | <0.0001 | |
| Deviance/df | 1.781 | | | |  | | 1.089 | | | | |  |
| LL | -6610.496 | | | |  | | -5667.230 | | | | |  |
| LL χ^2^ | 1579.017 | | | |  | | 799.779 | | | | |  |
| AIC | 13264.992 | | | |  | | 11378.461 | | | | |  |
| BIC | 13409.932 | | | |  | | 11523.402 | | | | |  |

Abbreviations: LL, Likelihood Ratio; AIC, Akaike information criterion, BIC, Bayesian information criterion

**- Description of data:**

We performed both Poisson and negative binomial regression analyses. AIC and BC are indexes for evaluating better models in the regression model with Poisson regression analysis and negative binomial regression as the same dependent variable and independent variable. The smaller this index is, the better the model. We used the following reference for how to select a good model: Posada, David, and Thomas R. Buckley. "Model selection and model averaging in phylogenetics: advantages of Akaike information criterion and Bayesian approaches over likelihood ratio tests." *Systematic biology* 53.5 (2004): 793-808. Finally, in the manuscript, we selected a negative binomial regression model as shown in Table 4.
