# Supplementary material for: The effects of continuity of care on hospital utilization in patients with knee osteoarthritis: analysis of Nationwide insurance data
Source: BMC Health Serv Res. 2018 Mar 2;18:152. doi: 10.1186/s12913-018-2951-y (PMC5833114; doi:10.1186/s12913-018-2951-y)
Supplement: Supplementary file 4 — Reduction in hospitalization and medical costs, according to the level of continuity of care (DOCX 19 kb) [file 12913_2018_2951_MOESM4_ESM.docx]

**- File name: Additional file 4**

**- Title of data:** Reduction in hospitalization and medical costs according to the level of continuity of care

|  | |  | COC index | | | |  |
| --- | --- | --- | --- | --- | --- | --- | --- |
|  |  | 0.76–1.00 | | 0.51–0.75 | 0.26–0.50 | 0.00–0.25 | |
| Number | | 78012 | 57320 | | 1470 | 23534 | |
| Hospitalization (N) | Incidence (%) | 0.6 | 4.6 | | 7.1 | 7.0 | |
|  | Mean (SD) | 0.007 (0.106) | 0.066 (0.384) | | 0.129 (0.615) | 0.101 (0.454) | |
|  | Difference† | 6.6% (=7.0%-0.6%) | | | | | |
|  | Reduction‡ | 3,566 (=6.6% × 23, 534) | | | | | |
|  | Conversion§ | 118,739 (=3566×100/3) | | | | | |
| Medical cost ($)* | Total cost* | 13,269,361 | 22,236,808 | | 297,230 | 11,515,441 | |
|  | Mean (SD) | 170 (312) | 388 (1041) | | 202 (627) | 489 (1158) | |
|  | Difference† | $319 (=489–170) | | | | | |
|  | Reduction‡ | 7,507,346 (=$319 × 23,534) | | | | | |
|  | Conversion§ | 249,994,621 (=7,507,346×100/3) | | | | | |
| * Costs are in Korean Won (1,200 KRW = 1 US dollar). † Difference = The incidence or mean cost of patients in the 0.00-0.25 COC index group - The incidence or mean cost of patients in the 0.76–1.00 COC index group ‡ Reduction = The difference of hospitalization incidence (%) or medical cost ($) × The number of patients in the 0.00-0.25 COC index group  §. Conversion to total population = Reduction in hospitalization or medical costs × Weight | | | | | | | |

**- Description of data:**

In order to analyze the extent to which continuous management reduces the burden of admission and medical expenses in Korea, the results of estimating the difference in medical burden between the highest and lowest sustainability indicators are shown in Additional file 4.
